# Regorafenib combined with transarterial chemoembolization for unresectable hepatocellular carcinoma: a real-world study

**DOI:** 10.1186/s12876-021-01967-3

**Published:** 2021-10-20

**Authors:** Yue Han, Guang Cao, Bin Sun, Jian Wang, Dong Yan, Haifeng Xu, Qinsheng Shi, Zechuan Liu, Weihua Zhi, Liang Xu, Bojun Liu, Yinghua Zou

**Affiliations:** 1 Department of Interventional Therapy, National Cancer Center, National Clinical Research Center for Cancer, Cancer Hospital, Chinese Academy of Medical Sciences and Peking Union Medical College, Beijing, 100021 China; 2 Key Laboratory of Carcinogenesis and Translational Research (Ministry of Education/Beijing), Department of Interventional Therapy, Peking University Cancer Hospital and Institute, Beijing, 100142 China; 3 Center of Interventional Oncology and Liver Diseases, Beijing Youan Hospital, Capital Medical University, Beijing, 100069 China; 4 Department of Interventional Radiology and Vascular Surgery, First Hospital, Peking University, Beijing, 100034 China

**Keywords:** Transarterial chemoembolization, Unresectable, Hepatocellular carcinoma, Regorafenib, Survival

## Abstract

**Background:**

The benefits and tolerability of transarterial chemoembolization (TACE) combined with regorafenib as a second-line therapy has not been reported for unresectable hepatocellular carcinoma (HCC). This study aimed to explore the benefits and tolerability of TACE combined with second-line regorafenib in patients with unresectable advanced HCC and failure to first-line treatment.

**Methods:**

This was a multicenter retrospective study of patients with progression after first-line sorafenib and/or lenvatinib between 01/2019 and 04/2020 at four tertiary hospitals in China. The patients were treated with TACE. Then, 5–7 days after the first TACE, the patients started taking regorafenib for 3 weeks every 4-week cycle. The overall survival (OS), time to progression (TTP), progression-free survival (PFS), and adverse events (AEs) were observed.

**Results:**

The median follow-up was 5.6 (range 0.7, 17.0) months. The median age was 60 (range 35, 79) years. There were 32 (84.2%) males. The patients underwent a median of three TACE sessions (range 1–13). The initial doses of regorafenib were 20 mg/d (n = 1, 2.6%), 80 mg/d (n = 10, 26.3%), 120 mg/d (n = 15, 39.5%), and 160 mg/d (n = 11, 28.9%). The incidence of grade 3/4 AEs was 15.8%. Two patients stopped regorafenib due to AEs. The median OS was 14.3 months. The median PFS and TTP were 9.1 (95% CI 4.0, 14.2) and 9.1 (95% CI 5.5, 12.8) months, respectively.

**Conclusions:**

The present study provides real-world evidence indicating that regorafenib combined with TACE was beneficial and tolerable in patients with unresectable HCC. Additional prospective large-scale studies are required for confirmation.

**Supplementary Information:**

The online version contains supplementary material available at 10.1186/s12876-021-01967-3.

## Background

Hepatocellular carcinoma (HCC) is a highly lethal invasive carcinoma arising in the liver and accounts for 75–85% of all liver cancers [1–4]. The worldwide age-standardized annual mortality rates of liver cancer are 13.9 per 100,000 in men and 4.9 per 100,000 in women [3]. The most important risk factors for HCC are infection with hepatitis B or hepatitis C and/or preexisting liver cirrhosis, and the incidence of HCC generally follows the geographical distribution of hepatitis B and C viruses [1, 5, 6]. In China, liver cancer ranks fourth among the most common malignant tumors and the second among the causes of cancer death [7, 8]. Due to the insidious onset of liver cancer, the symptoms are not obvious or typical, and most patients are diagnosed with advanced disease [1, 5, 6, 9, 10]. In addition, patients with advanced-stage HCC often cannot undergo radical treatment (surgical excision or liver transplantation) due to the status of liver function, shortage of donor liver source, and metastasis [1, 4, 6, 11–13].

Transarterial chemoembolization (TACE) and/or systemic drugs (such as sorafenib as first-line treatment) are recommended to treat inoperable patients with advanced HCC [1, 4, 6, 12]. Nevertheless, the efficacy of TACE alone is relatively poor, with complete response rates of only 0–4.8% [14]. The objective response rate (ORR) of TACE alone is lower than TACE combined with systemic treatment [15], and tumors still progress even after multiple TACE. Nevertheless, the efficacy of systemic treatment alone is also relatively poor [16–18]. Sorafenib, combined with TACE, has been proven to yield good outcomes [19]. Many studies confirmed that the efficacy of sorafenib combined with TACE in the treatment of advanced HCC is better than that of either TACE and antiangiogenic treatments alone, with an acceptable safety profile [15, 19–21].

Regorafenib (Bay 73-4506) is an oral multikinase inhibitor whose antitumor effect is achieved by blocking and inhibiting the activity of multiple protein kinases involved in tumor angiogenesis, tumorigenesis, metastasis, and tumor immunity [22, 23]. The international, multicenter, placebo-controlled phase III RESORCE trial confirmed that compared with placebo, patients with advanced HCC receiving regorafenib treatment had higher ORR and disease control rate (DCR) and longer progression-free survival (PFS), time to progression (TTP), and overall survival (OS) [24]. Therefore, regorafenib has become a standard second-line treatment for HCC after sorafenib treatment.

Since many studies confirmed the efficacy of sorafenib combined with TACE [15, 19–21], we hypothesized that second-line regorafenib combined with TACE might have better efficacy in patients with HCC who failed to undergo first-line TACE with or without sorafenib or rivatinib. Therefore, this multicenter retrospective study was conducted to explore the benefits and tolerability of TACE combined with second-line regorafenib in patients with unresectable advanced HCC and failure to first-line treatment.

## Methods

### Patients

This multicenter retrospective study included patients with progression after first-line sorafenib and/or lenvatinib between January 2019 and April 2020 at four tertiary hospitals in China. All experiments were performed in accordance with the Declaration of Helsinki. This study was approved by Chinese Ethics Committee of Registering Clinical Trials (ChiECRCT20210138). The requirement for individual informed consent was waived by Chinese Ethics Committee of Registering Clinical Trials.

The inclusion criteria were: (1) > 18 years of age; (2) pathologically or clinically diagnosed HCC[25]; (3) unresectable or refused surgery; (4) first-line sorafenib and/or lenvatinib treatment; (5) Eastern Cooperative Oncology Group (ECOG) performance status (PS) score of 0–2; (6) Barcelona clinic liver cancer (BCLC) stage B-C; (7) Child–Pugh grade A or B; (8) TACE; and (9) underwent TACE combined with regorafenib. The exclusion criteria were: (1) incomplete data; (2) complicated with other serious diseases, including severe cardiovascular and cerebrovascular diseases, severe liver function injury (2 times higher than reference value), severe renal injury (eGFR < 45 ml/min/1.73 m^2^), and hematologic disease; (3) history of other malignant tumors; (4) pregnant or lactating women; or (5) participation in a clinical trial.

### Treatment

The patients were treated with TACE. Then, 5–7 days after the first TACE, the patients started taking regorafenib for 3 weeks every 4-week cycle.

### Data collection and definitions

All data were collected from the electronic health records, including age, sex, body mass index (BMI), complications, pathological diagnosis ratio, liver cirrhosis, child–Pugh, ECOG score, BCLC stage, macrovascular invasion, extrahepatic spread, α-fetoprotein (AFP), hepatitis B/C infection, number of intrahepatic lesions, maximum lesion diameter, and previous treatments. The treatment-related variables were treatment time, initial dosage, dosage adjustment, course of treatment, reasons for withdrawal, time, method, frequency of TACE treatment, and reasons for termination of treatment.

The primary outcomes were PFS, TTP, and OS, calculated from the first TACE treatment. The secondary outcomes were complete response (CR), partial response (PR), progressive disease (PD), stable disease (SD), and ORR (according to mRECIST), drug safety (CTCAE 4.03), and surgical complications (Clavien classification). The last follow-up was on June 15, 2020.

### Statistical analysis

The continuous variables with a normal distribution were expressed as means ± standard deviations, and those with a skewed distribution were expressed as medians (ranges). Categorical variables were expressed as n (%). Survival analysis was performed using the Kaplan–Meier method and the log-rank test. Univariable and multivariable Cox proportional hazards models were used to analyze the prognostic factors. The variables with P-values < 0.05 in the univariable analyses were included in the multivariable analysis using the forward method. Two-sided P-values < 0.05 were considered statistically significant.

## Results

### Characteristics of the patients

A total of 38 patients were included in the study. The median follow-up was 5.6 (range 0.7, 17.0) months. The median age was 60 (range 35, 79) years. There were 32 (84.2%) males. Eighteen (47.4%) patients were in BCLC stage B and 20 (52.6%) were in BCLC stage C. ECOG performance status was 0 in 20 (52.6%) patients, 1 in 13 (34.2%), and 2 in five (13.2%), respectively. Regarding the Child–Pugh stage, one (2.6%) patient was grade B, and the others were grade A. There were 12 (31.6%) patients with macrovascular invasion and 13 (34.2%) with extrahepatic metastases. Previous treatment included surgery in 13 (34.2%) patients, TACE in 36 (94.7%), and radiation therapy in six (15.8%). Sorafenib was the main drug for previous systemic treatment, which was used in 33 (86.8%) patients (Table 1).

**Table 1:** Characteristics of the patients

Characteristic	Total (n = 38)
*Age (years)*	59.4 ± 9.2
Median (range)	60 (35, 79)
*Sex, n (%)*	
Male	32 (84.2)
Female	6 (15.8)
*ECOG performance status, n (%)*	
0	20 (52.6)
1	13 (34.2)
2	5 (13.2)
*BCLC grade, n (%)*	
B	18 (47.4)
C	20 (52.6)
*Hepatitis, n (%)*	
Hepatitis B	32 (84.2)
Hepatitis C	2 (5.3)
No	4 (10.5)
Maximum tumor size (cm)	3.75 (0.9, 15.1)
*Tumor number, n (%)*	
Single	4 (10.5)
Multiple	32 (84.2)
Unknown	2 (5.3)
*Child–Pugh grade, n (%)*	
A	37 (97.4)
B	1 (2.6)
Macrovascular invasion, n (%)	12 (31.6)
AFP ≥ 400 μg/L, n (%)	11 (28.9)
*Comorbidity, n (%)*	
Hypertension	15 (39.5)
Diabetes Mellitus	4 (10.5)
Extrahepatic metastasis, n (%)	13 (34.2)
*Prior local therapy, n (%)*	
Surgery	13 (34.2)
Ablation	20 (52.6)
TACE	36 (94.7)
Radiotherapy	6 (15.8)
*Prior systemic therapy, n (%)*	
Sorafenib	33 (86.8)
Lenvatinib	1 (2.6)
Lenvatinib sequential to sorafenib	4 (10.5)
Follow-up (months), median (range)	5.6 (0.7, 17.0)

*ECOG* Eastern Cooperative Oncology Group, *BCLC* Barcelona clinic liver cancer, *AFP* α-fetoprotein, *TACE* transarterial chemoembolization

### Treatment profile of TACE combined with regorafenib

Patients were treated with cTACE (n = 20, 52.6%) or D-TACE (n = 17, 44.7%), for a median of 3 (range 1, 13) sessions. The initial doses of regorafenib were 20 mg/d (n = 1, 2.6%), 80 mg/d (n = 10, 26.3%), 120 mg/d (n = 15, 39.5%), and 160 mg/d (n = 11, 28.9%). During treatment, 15 patients stopped regorafenib because of progression (n = 9, 60.0%), intolerance (n = 5, 33.3%), and others (n = 1, 6.1%) (Table 2).

**Table 2:** Treatment profile

Variable	Total (n = 38)
*TACE method, n (%)*	
cTACE	20 (52.6%)
D-TACE	17 (44.7%)
Unknown	1 (2.6%)
TACE sessions, n (range)	3 (1, 13)
*Regorafenib initial dosage, n (%)*	
20 mg/d	1 (2.6%)
80 mg/d	10 (26.3%)
120 mg/d	15 (39.5%)
160 mg/d	11 (28.9%)
Unknown	1 (2.6%)
Stopped regorafenib	15 (39.5%)
*Reasons for stopping*	
Progression	9 (23.7%)
Intolerance	5 (13.2%)
Other	1 (2.6%)

*ECOG* Eastern Cooperative Oncology Group

### Outcomes

**Fig. 1:**
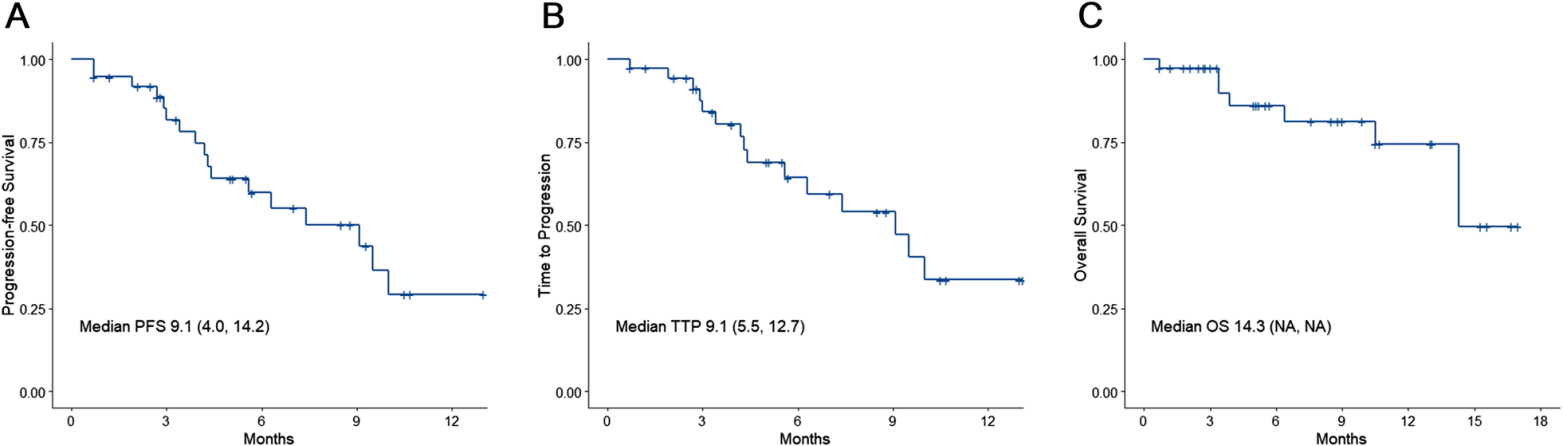
Kaplan–Meier plots of median **A** progression-free survival (PFS), **B** time-to-progression (TTP), and **C** overall survival (OS) in patients treated with TACE plus regorafenib

At the last follow-up, 15 patients experienced a progression, and seven patients had died. The best treatment responses were CR in one patient (2.6%), PR in two (5.3%), SD in 26 (68.4%), and PD in nine (23.7%), for an ORR of 7.9% and a DCR of 76.3% (Table 3). The median PFS was 9.1 (range: 4.0, 14.2) months, the median TTP was 9.1 (range 5.5, 12.7) months, and the median OS was 14.3 (NA, NA) months (Fig. 1).

**Table 3:** Tumor response

Variable	Total (n = 38)
*Best response, n (%)*	
Complete response	1 (2.6)
Partial response	2 (5.3)
Stable disease	26 (68.4)
Progressive disease	9 (23.7)
Objective response rate, n (%)	3 (7.9)
Disease control rate, n (%)	29 (76.3)
6-month PFS rate, % (95% CI)	59.8 (41.8, 77.8)

### Univariable and multivariable analyses

**Fig. 2:**
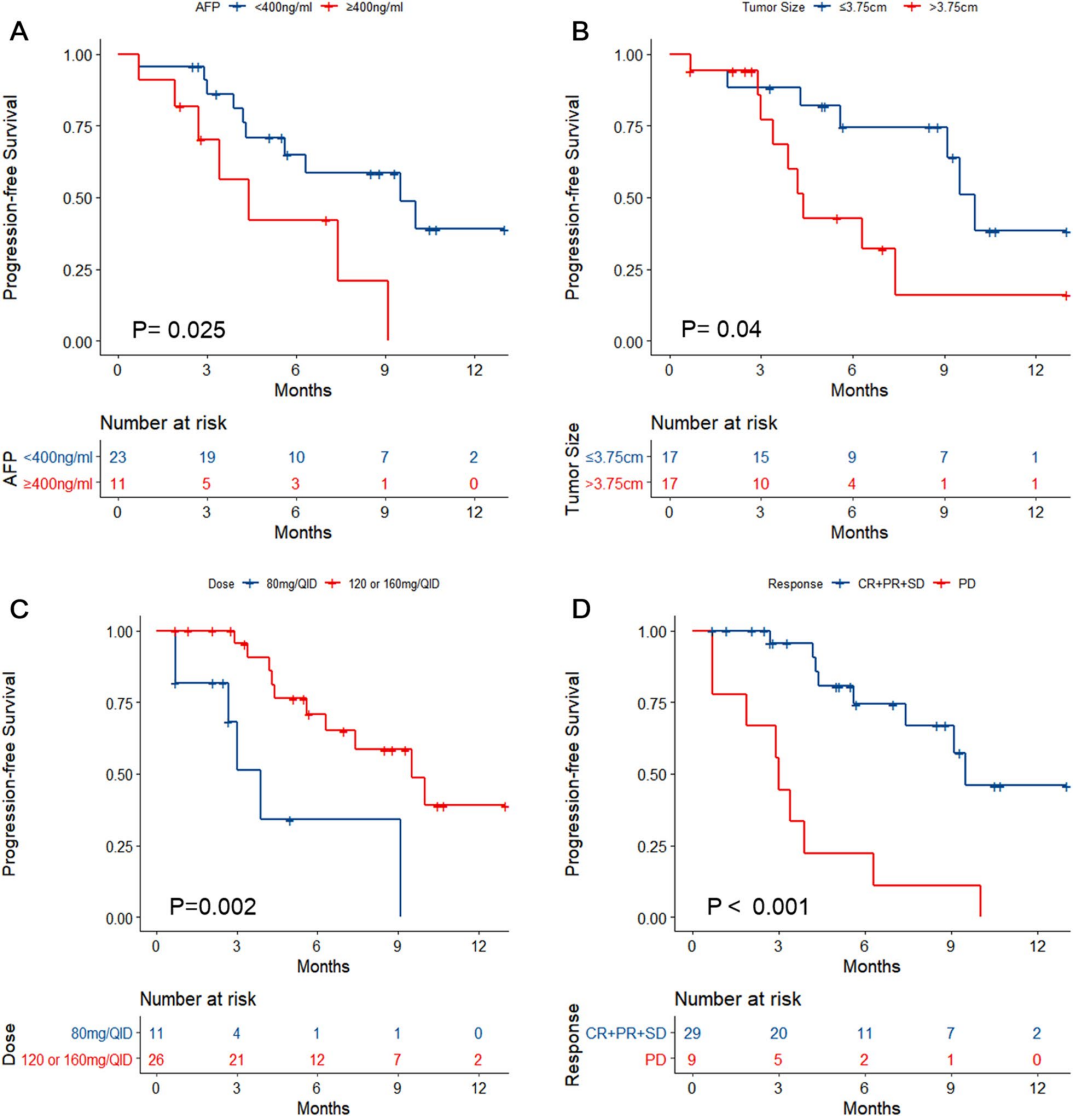
Kaplan–Meier analyses of progression-free survival according to **A** α-fetoprotein levels, **B** tumor size, **C** regorafenib dose, and **D** best response

Cox univariable analyses were performed with PFS as the outcome. AFP ≥ 400 ng/ml was associated with lower PFS than AFP < 400 ng/ml (log-rank P = 0.025; HR = 3.046, 95% CI 1.092–8.497, P = 0.033) (Fig. 2A and Additional file 1: Table S1). Tumor size > 3.75 cm was associated with PFS (log-rank P = 0.04; HR = 2.822, 95% CI 1.011–7.879, P = 0.048) (Fig. 2B and Additional file 1: Table S1). Initial dose of Regorafenib at 120 or 160 mg/d was associated with higher PFS than at 80 mg/d (log-rank P = 0.002; HR = 0.209, 95% CI 0.07–0.623, P = 0.005) (Fig. 2C and Additional file 1: Table S1). Patients achieving CR, PR, or SD had a better PFS than those with PD (log-rank P < 0.001; HR = 5.998, 95% CI 2.274–15.820, P < 0.001) (Fig. 2D and Additional file 1: Table S1).

After adjusting for AFP levels and tumor size in the Cox multivariable model, the initial dosage of 120 or 160 mg/d (HR = 0.216, 95% CI 0.061–0.765, P = 0.018) and patients achieving PD (HR = 5.607, 95% CI 1.896–16.578, P = 0.002) were independently associated with PFS (Additional file 1: Table S1).

**Fig. 3:**
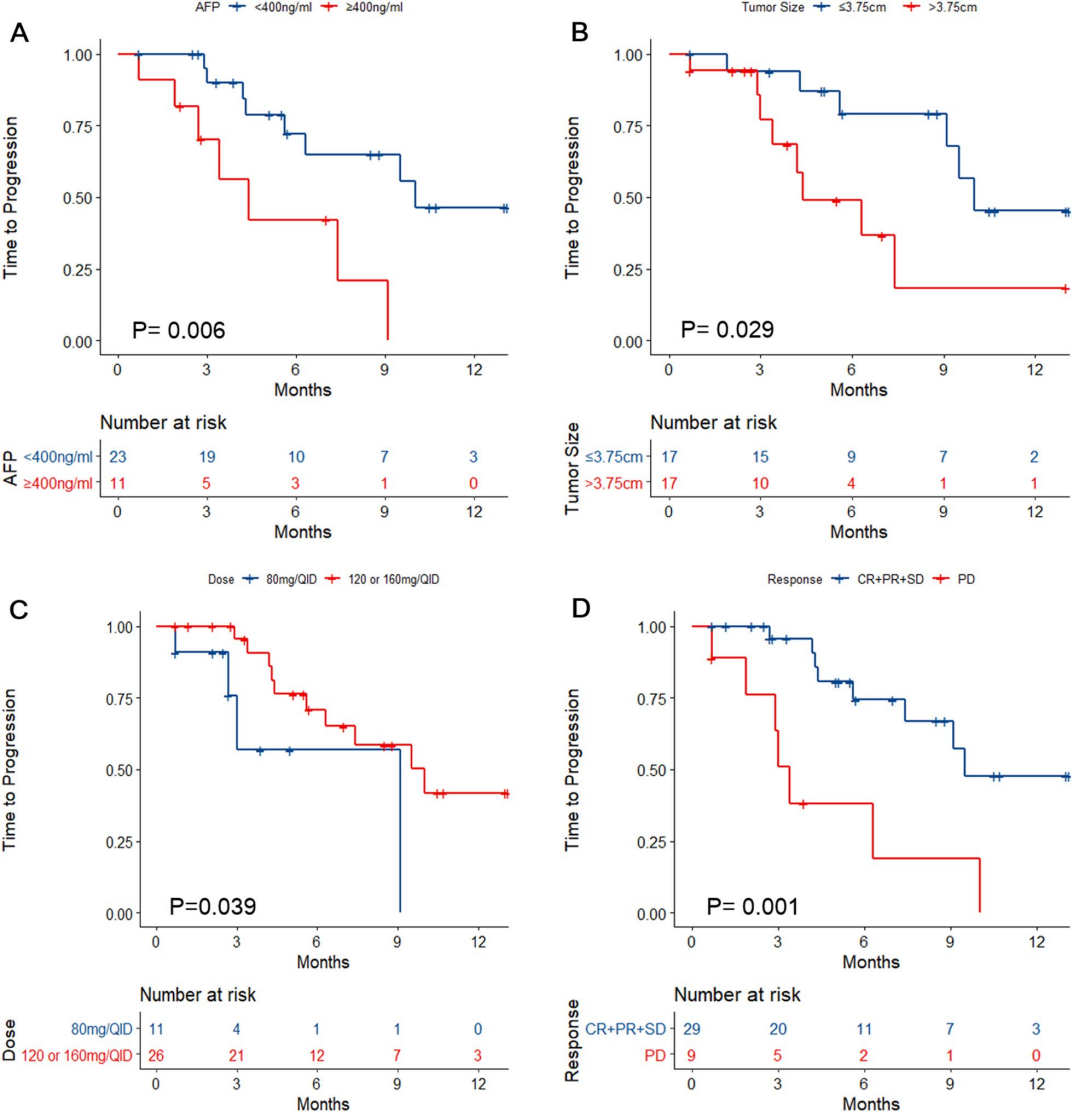
Kaplan–Meier analyses of time-to-progression according to **A** α-fetoprotein levels, **B** tumor size, **C** regorafenib dose, and **D** best response

Cox univariable analyses were performed with TTP as the outcome. AFP ≥ 400 ng/ml was associated with shorter TTP than AFP < 400 ng/ml (log-rank P = 0.006; HR = 4.19, 95% CI 1.391–12.596, P = 0.011) (Fig. 3A and Additional file 1: Table S2). Maximum tumor size > 3.75 cm was associated with shorter TTP (log-rank P = 0.029; HR = 3.252, 95% CI 1.077–9.821, P = 0.037) (Fig. 3B and Additional file 1: Table S2). Regorafenib at 120 or 160 mg/d was associated with longer TTP than at 80 mg/d (log-rank P = 0.039; HR = 0.293, 95% CI 0.085–1.006, P = 0.051) (Fig. 3C and Additional file 1: Table S2). Patients achieving CR, PR, or SD had a longer TTP than those with PD (log-rank P = 0.001; HR = 4.858, 95% CI 1.731–13.635, P = 0.003) (Fig. 3D and Additional file 1: Table S2).

After adjusting for tumor size in the Cox multivariable model, a maximum tumor size > 3.75 cm (HR = 5.544, 95% CI 1.616–19.02, P = 0.006) and patients achieving PD (HR = 7.691, 95% CI 2.37–24.964, P = 0.001) were independently associated with TTP (Additional file 1: Table S2).

**Fig. 4:**
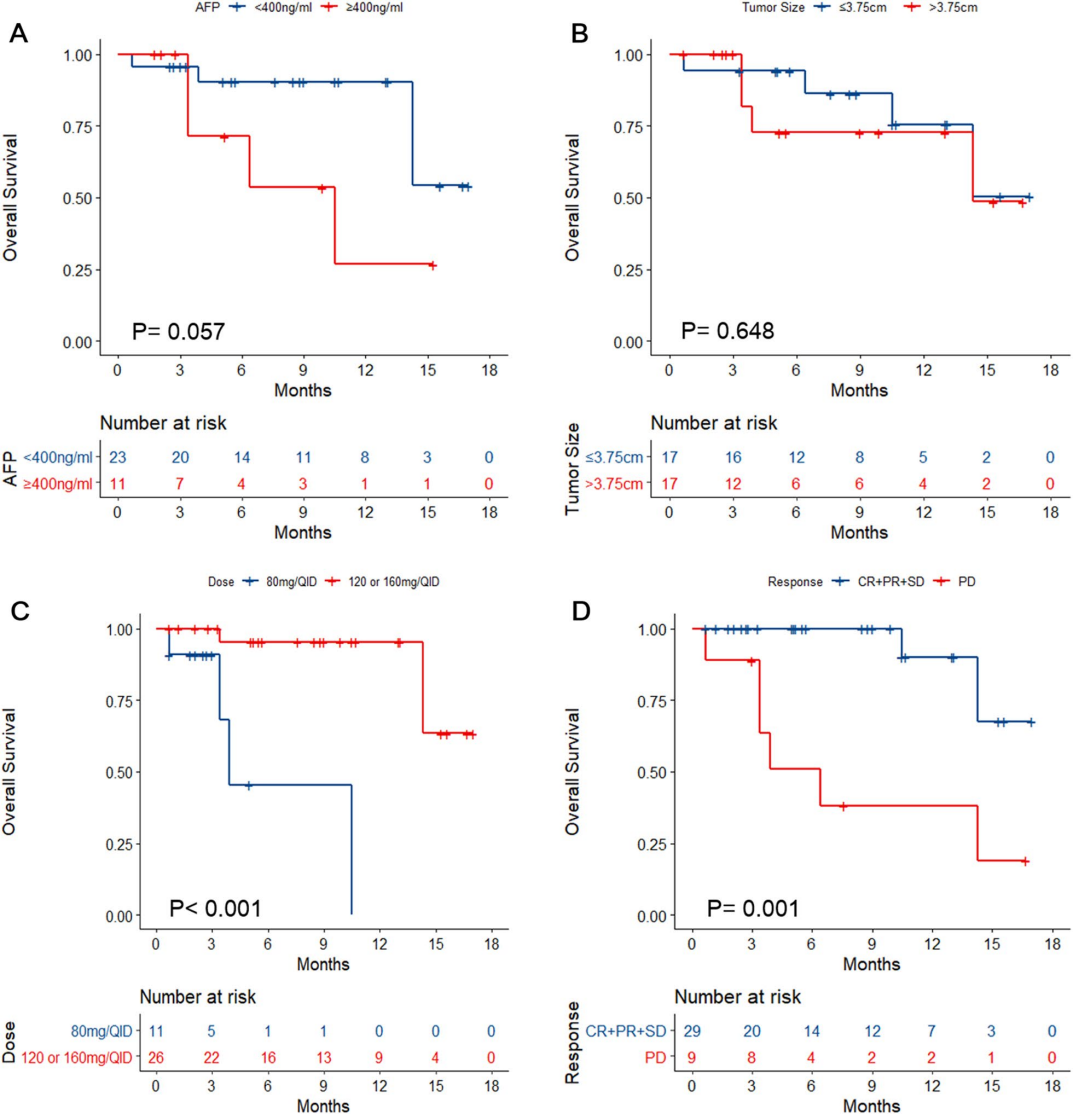
Kaplan–Meier analyses of overall survival according to **A** α-fetoprotein levels, **B** tumor size, **C** regorafenib dose, and **D** best response

Finally, Cox univariable analyses were performed with OS as the outcome. AFP was not associated with OS (log-rank P = 0.057; HR = 3.487, 95% CI 0.857–14.196, P = 0.081) (Fig. 4A and Additional file 1: Table S3). Tumor size was not associated with OS (log-rank P = 0.648; HR = 1.37, 95% CI 0.338–5.55, P = 0.66) (Fig. 4B and Additional file 1: Table S3). Regorafenib at 120 or 160 mg/d was associated with longer OS than at 80 mg/d (log-rank P < 0.001; HR = 0.043, 95% CI 0.005–0.397, P = 0.006) (Fig. 4C and Additional file 1: Table S3). Patients achieving CR, PR, or SD had a longer OS than those with PD (log-rank P = 0.001; HR = 9.151, 95% CI 1.823–45.939, P = 0.007) (Fig. 4D and Additional file 1: Table S3).

In the Cox multivariable model, an initial dosage at 120 or 160 mg/d (HR = 0.049, 95% CI 0.004–0.535, P = 0.013) and patients achieving PD (HR = 6.497, 95% CI 1.103–38.284, P = 0.039) were independently associated with OS (Additional file 1: Table S3).

### Toxicity

As shown in Table 4, no grade 5 AEs (death) were observed. After TACE, the complications included nausea (n = 11, 28.9%), pain (n = 11, 28.9%), vomiting (n = 3, 7.9%), and fever (n = 4, 10.5%). Seven (18.4%) patients experienced at least one grade 3–4 AE. There were two cases of drug withdrawal due to AEs. The most common AEs (≥ 10% occurrence) were hand-foot syndrome (n = 8, 21.1%), anemia (n = 4, 10.5%), leukopenia (n = 4, 10.5%), thrombocytopenia (n = 5, 13.2%), and elevated aspartate transaminase levels (n = 4, 10.5%).

**Table 4:** Postoperative complications and adverse events

	Total (n = 38)
*Postoperative complications*	
Nausea	11 (28.9%)
Pain	11 (28.9%)
Vomiting	3 (7.9%)
Fever	4 (10.5%)
*Adverse events*	
Grade 3–4 adverse events	7 (18.4%)
Hand-foot syndrome	8 (21.1%)
Anemia	4 (10.5%)
Leukopenia	4 (10.5%)
Thrombocytopenia	5 (13.2%)
Elevated aspartate transaminase	4 (10.5%)
Hyperbilirubinemia	2 (5.3%)
Diarrhea	4 (10.5%)
Nausea	1 (2.6%)
Hypertension	2 (5.3%)
Rash	2 (5.3%)

## Discussion

To the best of our knowledge, this study is the first to explore the feasibility, prognosis, and toxicity of TACE combined with regorafenib to treat patients with unresectable HCC and failure to first-line treatment. The results showed promising DCR, 6-month PFS rate, and median OS, and the treatment was tolerable. Patients who achieved disease control after TACE combined with regorafenib might have a better prognosis. TACE combined with regorafenib might be an alternative treatment for unresectable HCC after failure to first-line treatment.

The patients included in this study were those who received first-line targeted therapy for unresectable HCC. Of them, most were treated with TACE (94.7%), followed by ablation (52.6%) and surgery (34.2%). The median number of TACE sessions was 3 (1,13). Among the patients, 14 received sorafenib, five received ranvatinib, and four received both. Higher numbers of early treatments might increase the risk of adverse reactions. Nevertheless, in this study, 13.2% of the patients had drug withdrawal due to intolerance, which was lower than the 25% observed in the RESORCE study [24]. In addition, flexible treatment, such as lower initial dosage and on-demand TACE, might improve tolerance and should be explored.

In this study, TACE combined with regorafenib was used as a second-line treatment, leading to a median PFS of 9.1 (4.0, 14.2) months, median TTP of 9.1 (5.5, 12.7) months, and median OS of 14.3 (NA, NA) months. In the RESORCE study, regorafenib monotherapy was used in advanced HCC patients after sorafenib, with a median PFS of 3.1 months (95% CI 2.8–4.2), median TTP of 3.2 months (95% CI 2.9–4.2), and a median OS of 10.6 months (95% CI 9.1–12.1) [24]. Lee et al. [26] used regorafenib monotherapy after progression to sorafenib, and the median PFS was 2.7 months (95% CI 2.5–2.9 months), the median TTP was 2.6 months (95% CI 2.4–2.8 months), and the median OS was 10.0 months (95% CI 8.4–11.6 months) [26]. Compared with monotherapy, TACE combined with systemic therapy (using regorafenib after sorafenib treatment) might lead to longer PFS, TTP, and OS, but this will have to be confirmed.

The DCR in this study was 76.3%, higher than in the RESORCE study (65%) and the real-world study by Lee et al. [26] (34.8%). TACE is a regional therapy combined with targeted chemotherapy and arterial embolization [14], but TACE alone shows poor efficacy. Indeed, the hypoxic state induced by TACE stimulates tumor angiogenesis to bypass the blocked tumor feeding arteries to promote disease progression or metastasis [27, 28]. Incomplete tumor necrosis in the target area could also be involved [29]. Due to the rich blood supply of tumors, complex blood supply arteries, and poor arteriole opening [19, 22, 23], there are often residual tumor-supporting vessels after TACE.

Regorafenib is a systemic multikinase inhibitor. It can inhibit tumor angiogenesis by inhibiting vascular endothelial growth factor receptor (VEGFR)-1, VEGFR-2, VEGFR-3, receptor tyrosine-protein kinase (Tie-2), and other protein kinase activity and play a role in anti-angiogenesis [22–24, 26]. In addition, it can also exert multiple antitumor effects by inhibiting multiple kinases involved in tumor proliferation and tumor microenvironment [22, 23]. Therefore, the combination of TACE and regorafenib can achieve synergistic effects. In addition, although regorafenib and sorafenib have overlapping targets, regorafenib targets a wider range of kinases and has stronger inhibitory effects on VEGFR-2, PDGFR-β, FGFR-1, and c-Kit. At the same time, regorafenib can also inhibit Tie-2, which has a broader anti-angiogenesis effect [22, 30]. Therefore, in TACE combined with systemic therapy, regorafenib can be used after progression to sorafenib, with a better prognosis and higher response [24, 26].

The factors associated with the PFS, TTP, and OS in the univariable analyses were tumor diameter > 3.75 cm, AFP > 400 ng/ml, dose of regorafenib, and best response to regorafenib. Tumor size is an important staging and prognosis factor of HCC [1]. AFP levels indicate liver damage, and elevated AFP levels have been associated with a poor prognosis of HCC [1, 31, 32]. The dose of regorafenib was associated with the outcomes, with higher doses achieving better effects. Bruix et al. [24] only used the 160-mg/d dose, but the actual dose might vary in a real-world setting, and additional studies should examine this. Finally, of course, the patients who achieve CR, PR, or SD as their best response have a higher likelihood of longer survival and TTP.

There was no grade 5 AE in this study. The complications of TACE are well-known [15, 19, 28, 29], and no novel safety signal was observed. The incidence of grade 3–4 adverse events was 15.8%, much lower than in the RESORCE trial (67%) [24]. This lower frequency of AEs could be due to a reporting bias and a lack of active surveillance.

We agree that locoregional therapy in patients with metastatic disease is not necessarily the standard of care in cancer in general. Still, the literature contains several patients in whom treating the primary disease led to a better prognosis despite distant metastasis [33–37]. Indeed, death due to metastasis occurs when the affected organs cease their normal functions. Still, in HCC, liver dysfunction or gastrointestinal hemorrhage leading to death might occur before the dysfunction of other organs if the liver disease is left untreated [33, 38–44]. Therefore, managing the primary disease might improve the prognosis of these patients [33–37]. We are the first to agree that this approach can be appropriate only in selected patients, but we need data to determine who these patients are. Sharing these data is the prerequisite for science advancement in the field of HCC management.

There was no control group, which is a limitation of the study. Still, the present study provides data about TACE with regorafenib, which could be used as a basis for future trials. Yoo et al. [45] observed no differences among different doses of regorafenib on prognosis, while the present study observed a better prognosis with higher doses. It was a real-world study, not a randomized one, and hence the conclusions must be taken with caution because of the risk of bias for uncontrolled parameters. The initial dose was chosen according to the patients’ weight, Child–Pugh grade, and ECOG performance status.

## Conclusions

This retrospective study is limited by its small sample size and short follow-up time, making it difficult to analyze the long-term efficacy outcomes. Despite its retrospective nature, the present study provides real-world evidence indicating that regorafenib combined with TACE was beneficial and tolerable in patients with unresectable HCC. Additional prospective large-scale studies are required for confirmation.

## Supplementary Information

**Additional file 1: Table S1.** Univariable and multivariable analysis of progression-free survival. **Table S2.** Univariable and multivariable analysis of time-to-progression. **Table S3.** Univariable and multivariable analysis of overall survival.

## Data Availability

The datasets used and/or analyzed during the current study are available from the corresponding author on reasonable request.

## Abbreviations

TACE: Transarterial chemoembolization
HCC: Hepatocellular carcinoma
OS: Overall survival
TTP: Time to progression
PFS: Progression-free survival
AEs: Adverse events
DCR: Disease control rate
ECOG: Eastern Cooperative Oncology Group
PS: Performance status
BCLC: Barcelona clinic liver cancer
BMI: Body mass index
AFP: α-Fetoprotein
CR: Complete response
PR: Partial response
PD: Progressive disease
SD: Stable disease
