# Supplementary material for: Regorafenib combined with transarterial chemoembolization for unresectable hepatocellular carcinoma: a real-world study
Source: BMC Gastroenterol. 2021 Oct 20;21:393. doi: 10.1186/s12876-021-01967-3 (PMC8529854; doi:10.1186/s12876-021-01967-3)
Supplement: Supplementary file 1 — Additional file 1: Table S1. Univariable and multivariable analysis of progression-free survival. Table S2. Univariable and multivariable analysis of time-to-progression. Table S3. Univariable and multivariable analysis of overall survival. [file 12876_2021_1967_MOESM1_ESM.docx]

**Table S1.** Univariable and multivariable analysis of progression-free survival

| Variable | Univariable | | | Multivariable | | |
| --- | --- | --- | --- | --- | --- | --- |
|  | HR | 95%CI | P | HR | 95%CI | P |
| Age (>60 vs. ≤60) | 1.649 | (0.627,4.341) | 0.311 |  |  |  |
| Hypertension (Yes vs. no) | 0.662 | (0.244,1.796) | 0.418 |  |  |  |
| ECOG score (1+2 vs. 0) | 1.364 | (0.512,3.636) | 0.535 |  |  |  |
| BCLCs stage (C vs. B) | 1.878 | (0.714,4.939) | 0.202 |  |  |  |
| Extrahepatic metastasis (Yes vs. no) | 1.471 | (0.541,4.002) | 0.449 |  |  |  |
| AFP (>400 vs. <400) | 3.046 | (1.092, 8.497) | **0.033** |  |  |  |
| Maximum tumor size (>3.75 vs. ≤3.75 cm) | 2.822 | (1.011,7.879) | **0.048** |  |  |  |
| Previous radical operation (Yes vs. no) | 0.737 | (0.268,2.028) | 0.554 |  |  |  |
| Previous ablation (Yes vs. no) | 0.853 | (0.317,2.295) | 0.754 |  |  |  |
| Previous radiotherapy (Yes vs. no) | 0.776 | (0.099,6.09) | 0.810 |  |  |  |
| TACE method (D-TACE vs. cTACE) | 1.885 | (0.698,5.087) | 0.211 |  |  |  |
| TACE (>3 vs. <3) | 0.989 | (0.363,2.692) | 0.983 |  |  |  |
| Initial dosage (160+120 vs. 80 mg/d) | 0.209 | (0.07,0.623) | **0.005** | 0.216 | (0.061,0.765) | **0.018** |
| Response (PD vs. CR+PR+SD) | 5.998 | (2.274, 15.820) | **<0.001** | 5.607 | (1.896,16.578) | **0.002** |

**Table S2.** Univariable and multivariable analysis of time-to-progression

| Variable | Univariable | | | Multivariable | | |
| --- | --- | --- | --- | --- | --- | --- |
|  | HR | 95%CI | P | HR | 95%CI | P |
| Age (>60 vs. ≤60) | 1.823 | (0.647,5.141) | 0.256 |  |  |  |
| Hypertension (Yes vs. no) | 0.598 | (0.205,1.743) | 0.346 |  |  |  |
| ECOG score (1+2 vs. 0) | 0.931 | (0.314,2.756) | 0.897 |  |  |  |
| BCLCs stage (C vs. B) | 1.566 | (0.558,4.394) | 0.394 |  |  |  |
| Extrahepatic metastasis (Yes vs. no) | 1.89 | (0.667,5.36) | 0.231 |  |  |  |
| AFP (>400 vs. <400) | 4.186 | (1.391, 12.596) | **0.011** | 5.544 | (1.616,19.02) | **0.006** |
| Maximum tumor size (>3.75 vs. ≤3.75 cm) | 3.252 | (1.077,9.821) | **0.037** |  |  |  |
| Previous radical operation (Yes vs. no) | 0.62 | (0.208,1.85) | 0.392 |  |  |  |
| Previous ablation (Yes vs. no) | 1.251 | (0.418,3.743) | 0.688 |  |  |  |
| Previous radiotherapy (Yes vs. no) | 0.041 | (0,368.343) | 0.492 |  |  |  |
| TACE method (D-TACE vs. cTACE) | 1.712 | (0.596,4.918) | 0.318 |  |  |  |
| TACE (>3 vs. <3) | 0.853 | (0.29,2.507) | 0.773 |  |  |  |
| Initial dosage (160+120 vs. 80 mg/d) | 0.293 | (0.085, 1.006) | 0.051 |  |  |  |
| Response (PD vs. CR+PR+SD) | 4.858 | (1.731,13.635) | **0.003** | 7.691 | (2.37,24.964) | **0.001** |

**Table S3.** Univariable and multivariable analysis of overall survival

| Variable | Univariable | | | Multivariable | | |
| --- | --- | --- | --- | --- | --- | --- |
|  | HR | 95%CI | P | HR | 95%CI | P |
| Age (>60 vs. ≤60) | 1.764 | (0.421,7.396) | 0.438 |  |  |  |
| Hypertension (Yes vs. no) | 0.341 | (0.069,1.692) | 0.188 |  |  |  |
| ECOG score (1+2 vs. 0) | 2.870 | (0.682,12.086) | 0.151 |  |  |  |
| BCLCs stage (C vs. B) | 1.408 | (0.347,5.721) | 0.632 |  |  |  |
| Extrahepatic metastasis (Yes vs. no) | 1.317 | (0.314,5.529) | 0.707 |  |  |  |
| AFP (>400 vs. <400) | 3.487 | (0.857,14.196) | 0.081 |  |  |  |
| Maximum tumor size (>3.75 vs. ≤3.75 cm) | 1.370 | (0.338,5.55) | 0.660 |  |  |  |
| Previous radical operation (Yes vs. no) | 1.475 | (0.363,5.999) | 0.587 |  |  |  |
| Previous ablation (Yes vs. no) | 0.462 | (0.11,1.934) | 0.290 |  |  |  |
| Previous radiotherapy (Yes vs. no) | 3.108 | (0.314,30.757) | 0.332 |  |  |  |
| TACE method (D-TACE vs. cTACE) | 2.720 | (0.657,11.252) | 0.167 |  |  |  |
| TACE (>3 vs. <3) | 0.501 | (0.1,2.513) | 0.401 |  |  |  |
| Initial dosage (160+120 vs. 80 mg/d) | 0.043 | (0.005,0.397) | **0.006** | 0.049 | (0.004,0.535) | **0.013** |
| Response (PD vs. CR+PR+SD) | 9.151 | (1.823,45.939) | **0.007** | 6.497 | (1.103,38.284) | **0.039** |
